# Canine Infections with *Onchocerca lupi* Nematodes, United States, 2011–2014

**DOI:** 10.3201/eid2105.141812

**Published:** 2015-05

**Authors:** Domenico Otranto, Alessio Giannelli, Maria S. Latrofa, Filipe Dantas-Torres, Nicole Scotty Trumble, Matt Chavkin, Gavin Kennard, Mark L. Eberhard, Dwight D. Bowman

**Affiliations:** Università degli Studi di Bari, Valenzano, Italy (D. Otranto, A. Giannelli, M.S. Latrofa, F. Dantas-Torres);; Aggeu Magalhães Research Institute, Recife, Brazil (F. Dantas-Torres);; BluePearl Veterinary Partners, Eden Prairie, Minnesota, USA (N. Scotty Trumble);; Veterinary Specialty and Emergency Hospital, Englewood, Colorado, USA (M. Chavkin);; Eye Care for Animals, Albuquerque, New Mexico, USA (G. Kennard);; Centers for Disease Control and Prevention, Atlanta, Georgia, USA (M.L. Eberhard);; Cornell University, Ithaca, New York, USA (D.D. Bowman)

**Keywords:** Onchocerca lupi, nematodes, roundworms, onchocercosis, dogs, canines, zoonoses, infections, ocular infestation, parasites, United States

## Abstract

Infections with *Onchocerca lupi* nematodes are diagnosed sporadically in the United States. We report 8 cases of canine onchocercosis in Minnesota, New Mexico, Colorado, and Florida. Identification of 1 cytochrome *c* oxidase subunit 1 gene haplotype identical to 1 of 5 from Europe suggests recent introduction of this nematode into the United States.

The number of human cases of zoonotic filariasis is increasing across industrialized countries (*1*). In particular, a major zoonotic potential has been recently recognized for *Dirofilaria immitis* and *D. repens* nematodes that infect dogs; both of these nematodes have been reported in cases of human dirofilariasis in the Western Hemisphere and the Old World (*1**,**2*).

After the first description of *Onchocerca lupi* nematodes in 1967 in a Caucasian wolf (*Canis lupis cubanensis*) from Georgia (former Union of Soviet Socialist Republics), this nematode has been recognized as the causative agent of canine and feline onchocercosis (*3**,**4*). In dogs, the infection occurs in an acute or chronic form characterized by ocular nodules that are often evident on the eyelids, conjunctiva, and sclera (*3**,**5*). However, if nematodes localize in the retrobulbar space of the eye, the infection may remain undetected (*6*). Nonetheless, in disease-endemic areas, *O. lupi* microfilariae may be isolated from skin sediments of apparently healthy dogs (*7*). Thus, dogs with overt ocular infections might represent only a small portion of the population in which canine onchocercosis occurs in countries such as Hungary, Greece, Germany, and Portugal (*3**,**7*).

The role of *O. lupi* nematodes as an agent of infection in dogs in the United States has been suspected. However, nematodes were previously identified only as *Onchocerca* sp. in California and Utah (*8**,**9*) or as *O. lienalis* in Arizona (*10*). Recent etiologic delineation of *O. lupi* nematodes in dogs and cats in southwestern states (*4**,**11**,**12*) suggested involvement of this parasite in previous cases.

After the first case report of human ocular onchocercosis caused by *O. lupi* nematodes in Turkey (*13*), interest in this parasite has been renewed, and additional zoonotic cases have been identified in Turkey, Tunisia, and Iran (*14*). In addition, this parasite has been extracted from the cervical channel of a 22-month-old child in Arizona (*12*). Information on the epidemiology and life history of *O. lupi* nematodes is still minimal, and data on its distribution in the United States is limited to 6 case reports (*4**,**11*).

We report 8 cases of *O. lupi* nematode infection in dogs from Minnesota, New Mexico, Colorado, and Florida. We also compare cytochrome *c* oxidase subunit 1 (*cox*1) gene sequences from 2 nematodes with sequences from parasites in Europe to determine possible recent introduction of this filarioid from Europe to the United States.

## The Study

**Figure 1:**
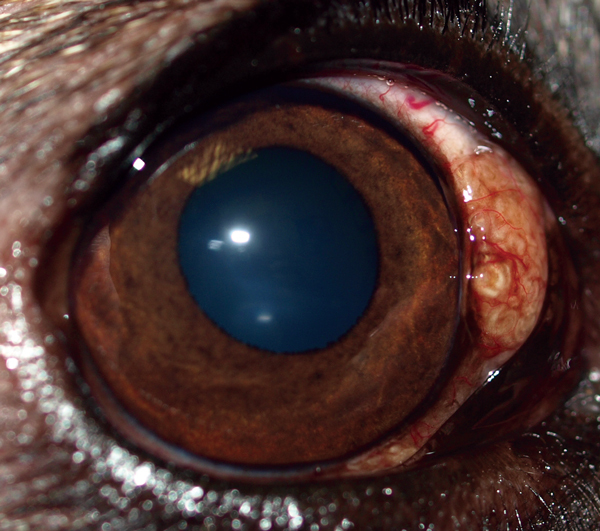
Subconjunctival nodule on the medial canthus of the right eye of dog 2 (Table), Minnesota, USA. This dog was found to be infected with *Onchocerca lupi* nematodes.

During April 2011–August 2014, a total of 8 privately owned dogs of various ages and sexes were referred to clinical practices in Minnesota (n = 1), New Mexico (n = 4), Colorado (n = 2), and Florida (n = 1) because of different degrees of ocular alterations (Table). At physical examination, nodules were detected in different areas of the eye (Figure 1) and associated with inflammatory reactions ranging from mild scleritis to episcleral swelling and vascular congestion (Table).

**Table:** Characteristics of 8 dogs infected with *Onchocerca lupi* nematodes, United States

Dog no.	Age, y/sex	Geographic origin (travel history)	Clinical signs
1	8/F	Hollywood, Florida (rescued)	Conjunctival and relapsing lesions in both eyes
2	7/M	Oronoco, Minnesota (Durango, Colorado)	Mild scleritis, proliferative eye lesions
3	6/F	Englewood, Colorado (Farmington, New Mexico)	Episcleral swelling and vascular congestion, squinting, scleral indentation in the temporal fundus
4	2/F	Englewood, Colorado (Farmington, New Mexico)	Inflammatory conjunctival follicles, mild epiphora, mild diffuse conjunctival hyperemia, episcleral
5	3/M	Farmington, New Mexico	Moderate blepharospasm and conjunctival hyperemia
6	3/M	Farmington, New Mexico	Moderate chemosis, episcleral flocculent mass
7	9/F	Jerez, New Mexico	Chronic waxing/waning episcleral mass
8	5/M	Farmington, New Mexico	Chronic conjunctivitis, superficial keratitis, episcleral mass, chemosis

All nodules were surgically removed from bulbar conjunctiva or sclera, and white filaria-like parasites were collected and stored in 70% ethanol for morphologic identification. In addition, specimens extracted from 2 dogs (dogs 2 and 3) (Table) were characterized by using molecular techniques. All dogs were treated with macrofilaricides, microfilaricides, antimicrobial drugs, and corticosteroids, which lead to complete resolution of ocular conditions in all except 3 animals (dogs 1, 7, and 8). These 3 dogs had relapses 2, 6, and 12 months, respectively, after surgery.

Nematodes had external, round, transverse ridges and 2 transverse striae per each outer ridge interval, which suggested that they were filarial worms of the genus *Onchocerca.* The ratio between body diameter and distances between ridges (7–10:1) was specific for *O. lupi* nematodes (*15*). A small piece of nematode was used for molecular identification. Genomic DNA was extracted and partial *cox*1 genes were amplified and sequenced as described (*13*).

In accordance with clinical signs of nodular ocular lesions and morphologic identification, partial *cox*1 gene sequence analysis (GenBank accession nos. KP283476 and KP283477) confirmed the identity of the nematode as *O. lupi,* showing 98% nt homology with other sequences of *O. lupi* nematodes in GenBank (KC686701 from Portugal and KC686702 from Greece) and 100% with those derived from dogs and cats from the United States, as well as with a sequence from Greece (EF521409).

**Figure 2:**
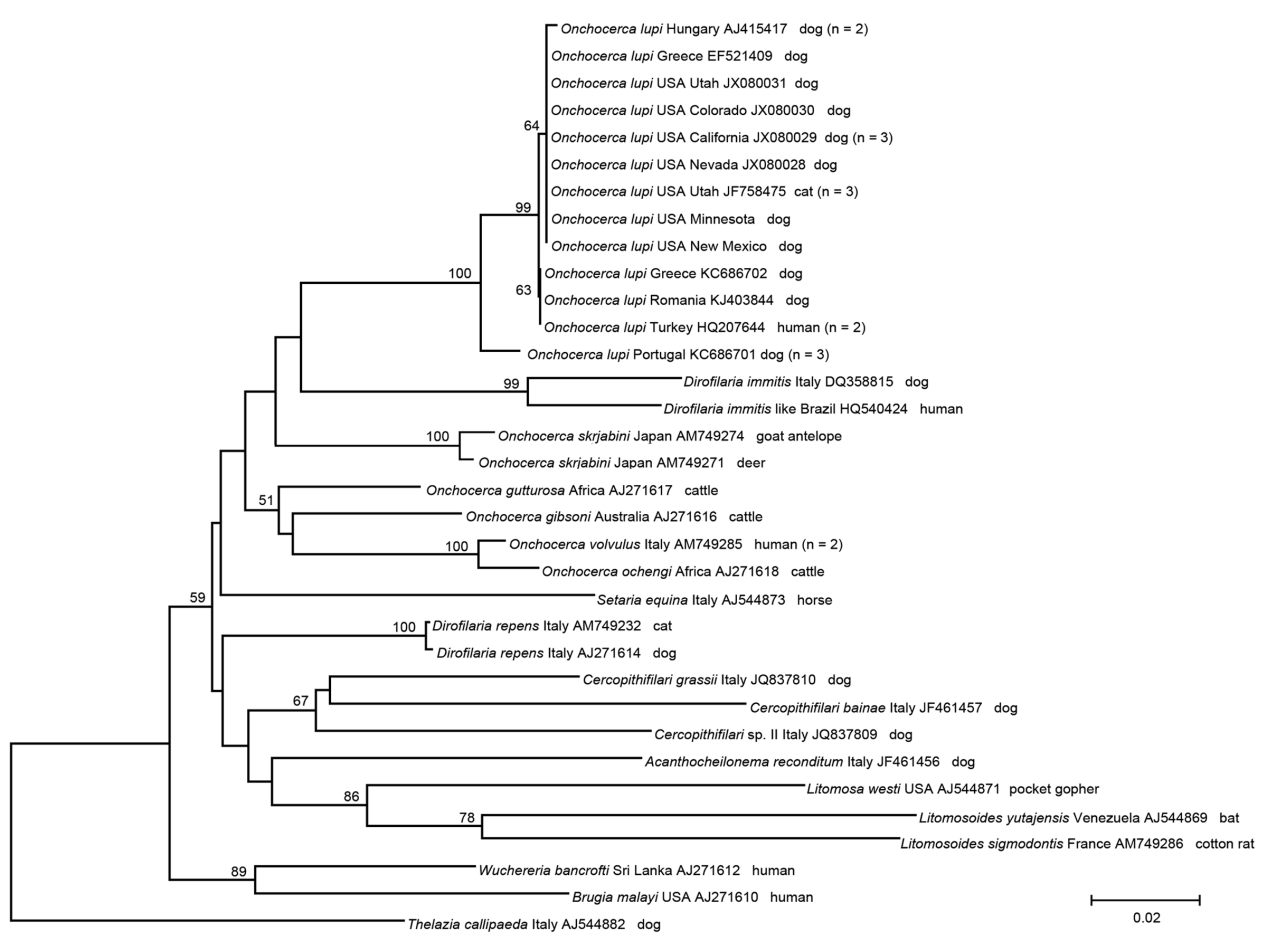
Phylogeny of *Onchocerca lupi* and other filarial nematode species based on partial sequences of the cytochrome *c* oxidase subunit 1 gene. *Thelazia callipaeda* was used as an outgroup. Bootstrap confidence values (values along branches) are for 8,000 replicates. GenBank accession numbers, number of haplotype sequences (values in parentheses), and geographic origins are shown. Scale bar indicates nucleotide substitutions per site.

Phylogenetic analysis of partial *cox*1 gene sequences was performed by using the neighbor-joining method and the Kimura 2-parameter model in MEGA5 (http://www.megasoftware.net/). This analysis confirmed that sequences from nematodes examined clustered with *O. lupi* sequences from different areas of the United States (Nevada, California, Colorado, Utah) and with a sequence from Greece (Figure 2). In addition, these sequences were grouped with others from Greece, Hungary and Portugal and formed a paraphyletic clade with other *Onchocerca* species available in GenBank.

## Conclusions

Our results indicate that a unique haplotype of *O. lupi* nematodes is circulating in the United States and is endemic to the canine population in this country. Although this onchocercid has been implicated as the causative agent of canine onchocercosis in the United States only recently (*11*), previous cases attributed to *Onchocerca* spp. have been described in dogs from Arizona, California, and Utah (*5**,**8**–**10*). The cases herein reported from Florida, New Mexico, and Minnesota suggest that the distribution of this nematode is probably wider than previously believed. Detection of *O. lupi* nematodes in Englewood, Colorado, confirms a previous report of infection in a dog from Mancos (*11*).

We identified 1 *cox*1 haplotype and found that it was identical to all sequences in GenBank from the United States and 1 from Greece. Conversely, up to 5 haplotypes were detected in Greece, Turkey, Iran, and Hungary (*7*). Genetic variation detected in *O. lupi* nematodes from Europe, Turkey, and Iran, along with isolation of this parasite from the Caucasian wolf, suggests that the infection probably originated in the Old World and was imported into the United States.

The low genetic distance detected for the *cox*1 gene is evidence of a substantially reduced evolutionary rate, which supports relatively recent divergence among specimens found in the Old World and New World. In addition to recent detection of *O. lupi* nematode infections in the United States, circulation of 1 haplotype could also suggest that a unique vector species occurs in areas of the Old World and New World where the infection has been diagnosed.

Given that all reports above are based on clinical signs, the epidemiology of *O. lupi* nematodes in the United States deserves to be thoroughly investigated. In particular, dogs relocated from disease-endemic areas to new areas should be routinely screened for skin-dwelling microfilariae because these parasites might represent a risk for other animals. In addition, because *O. lupi* nematodes circulate among canine populations, the potential role of dogs as reservoirs for human infection should not be underestimated, as also inferred by zoonotic cases reported in the United States (*12*). Finally, further studies are urgently warranted toward improving the diagnosis of *O. lupi* nematode infections, which will lead to a better appreciation of its distribution and potential risk for human populations.
